# Treatment outcomes and associated factors in severe malaria patients at University of Gondar Hospital, Northwest Ethiopia: A retrospective study (2020–2023)

**DOI:** 10.1371/journal.pone.0309681

**Published:** 2024-12-03

**Authors:** Marshet Anteneh, Mezgebu Silamsaw Asres, Geberehiwot Lema Legese, Meron Asmamaw Alemayehu, Dagmawi Woldesenbet, Desalew Getahun Ayalew

**Affiliations:** 1 Bahir-Dar Blood Bank, Amhara National Regional State Health Bureau, Bahir-Dar, Ethiopia; 2 Department of Internal Medicine, College of Medicine and Health Sciences, University of Gondar, Gondar, Ethiopia; 3 Department of Epidemiology and Biostatistics, Institute of Public Health, College of Medicine and Health Sciences, University of Gondar, Gondar, Ethiopia; 4 Department of Medical Laboratory Science, College of Medicine and Health Science, Wachemo University, Hossana, Ethiopia; Para Federal University, BRAZIL

## Abstract

**Background:**

Malaria continues to be the most prevalent life-threatening parasitic illness in Ethiopia. Its clinical spectrum ranges from mild to severe, with a propensity for death. In Ethiopia, it accounts for 10% of hospital admission. Identifying predictors of malaria-related mortality is crucial for aiding high-risk patient identification and enabling timely intervention.

**Objective:**

Our study aimed to assess treatment outcomes and factors associated with mortality among severe malaria patients at the University of Gondar Comprehensive Specialized Hospital, Northwest Ethiopia.

**Methods:**

A retrospective cross-sectional study examined 383 randomly chosen patients with severe malaria, spanning a four-year period leading up to the data collection date, encompassing July 2023 back to June 2020. Data were collected from the hospital records. A structured questionnaire was used to collect the data. EpiData version 3.1 and SPSS version 20 were used to clean and analyze the data, respectively. Logistic regression analysis was conducted to determine associations and reported by the odds ratio at p < 0.05 with 95% confidence intervals.

**Results:**

Among the 383 eligible patients, the majorities were males (56.66%) and resided in rural areas (66.32%). Over 84% of them were referred from health facilities. *Plasmodium falciparum* was the major parasite identified in 78% of cases. The magnitude of death among severe malaria patients was 10.97%. Impaired consciousness, convulsions, jaundice, parasitemia level >2, and creatinine level ≥3 were significantly associated with death, with adjusted odds ratios (AOR) of 3.4 (95% CI: 1.3–8.3), 2.7 (95% CI: 1.004–7.492), 3.2 (95% CI: 1.173–9.182), 3.7 (95% CI: 1.516–9.113), and 11.7 (95% CI: 4.756–29.239), respectively.

**Conclusion:**

Our study revealed a significant number of malaria-related deaths, with predictors such as age, impaired consciousness, convulsions, jaundice, parasitemia level, and creatinine level identified. Hence, it is imperative to implement intense and timely interventions for patients exhibiting these clinical manifestations to prevent malaria-related fatalities.

## Introduction

Malaria is the most common life-threatening parasitic disease in the world. It is caused by parasites of the genus *Plasmodium* and transmitted by the bite of an infected female *Anopheles* mosquito [1]. The disease is one of the leading causes of mortality and morbidity in many developing countries, with over 84 endemic countries and territories experiencing on-going malaria transmission (8). In 2022, the World Health Organization (WHO) reported that 249 million people are infected with malaria, resulting in 608,000 deaths worldwide [2].

In Ethiopia, malaria is a significant public health issue. Ethiopian Ministry of Health report states that at the end of 2019, there had been 4,782 recorded malaria deaths and 2.9 million confirmed cases [3]. In Ethiopia, 10% of admissions to medical facilities and 12% of outpatient consultations are made by malaria [4].

Malaria has a range of clinical presentations, from mild symptoms to severe and critical conditions with the possibility of progressing to death [1]. Severe malaria is often associated with cerebral involvement, pulmonary edema, Acute Kidney Injury (AKI), severe anemia, jaundice, shock and/or hemorrhage, as well as metabolic problems such as acidosis and hypoglycemia. Any of these problems might arise quickly and lead to death within hours or days [5].

Almost all severe malaria is caused by *Plasmodium (P) falciparum* and needs urgent medical attention. Other *Plasmodium* species seldom cause major complications or even death [6]. Acute respiratory distress syndrome (ARDS), cerebral malaria, jaundice, renal impairment, severe anemia, and impaired consciousness are just a few of the life-threatening complications that can result from severe malaria [7, 8]. Studies have identified several risk factors for severe malaria and death, including age over 65 years, female sex (especially during pregnancy), non-immune status, coexisting medical conditions, lack of antimalarial prophylaxis, delayed treatment, poor treatment adherence and severity of illness at admission (such as coma, ARI, shock, pulmonary edema, and coagulation disorders) [9–12]. The risk of death increases with the number of severe criteria present at presentation. Without prompt treatment, severe complications and deaths may occur within one week of fever onset [13].

In Ethiopia, vectors insecticide resistance and the parasites drug resistance, along with climatic changes, continue to pose challenges to the success of the national malaria elimination plan by 2030 [14]. Additionally, Gondar town and surrounding area are susceptible to seasonal adult migration to the highly malaria-prevalent Metema-Humera lowlands.

According to the WHO report, there were additional 5 million cases of malaria worldwide by 2022 as compared to the previous year. Ethiopia, which contributes 1.3 million malaria cases, was one of the primary countries to the increase in prevalence [15]. The Amhara region is affected more than other regions of the country, with 31% of the national malaria burden [16].

Despite the utilization of anti-malarial drugs and advanced medical interventions such as admission to an intensive care unit, mechanical ventilation, hemodialysis, and blood transfusion to treat severe malaria cases [17], the number of malaria-related deaths in Ethiopia remains fluctuating [18, 19]. Therefore, understanding the predictors of malaria-related death holds significant prognostic value, as it facilitates the prompt identification of high-risk patients, enables timely management, and encourages early referral to advanced healthcare institutions for better treatment. Hence, our study aimed to assess treatment outcomes and factors associated with mortality among severe malaria patients at University of Gondar Comprehensive Specialized Hospital (UoGCSH).

## Methods and materials

### Study setting and population

**Fig 1:**
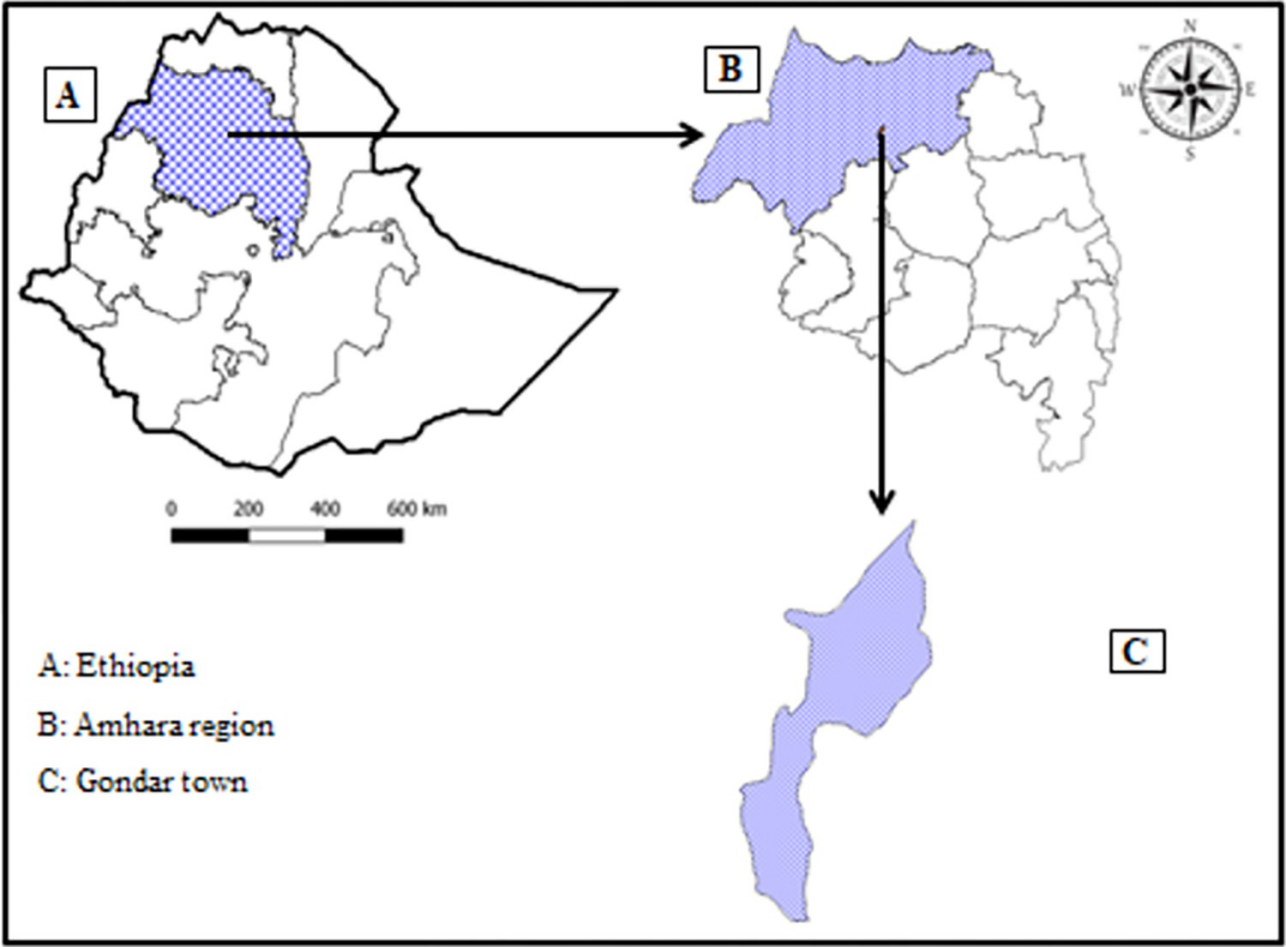


The study was conducted at UoGCSH, which is found in Gondar town. Gondar is one of the ancient cities in Ethiopia which is 720 km away from the capital city Addis Ababa, and 180 km from Bahir-Dar, the capital city of Amhara National Regional State (Fig 1). The latitude and longitude of Gondar is 12.6030° N, 37.4521° E is respectively. The hospital is one of the tertiary teaching hospitals in Northwest Ethiopia. It provides health care service for over 7 million people. Moreover, the hospital provides healthcare services for more than 60 years. Our study populations were all adult severe malaria patients fulfilling the WHO severe malaria criteria [20], and admitted to the hospital during 2020 to 2023.

### Eligibility criteria

The study excluded patient charts with incomplete medical records and those lacking microscopic laboratory data for malaria.

### Study design and period

We employed a retrospective cross-sectional study design to examine severe malaria patients registered over a four-year period, spanning from June 2020 to July 2023. Data retrieval occurred between August 1, 2023, and November 30, 2023.

### Operational definitions

- Admission outcome: the treatment outcome of the study i.e. either death or recovery.
- Comorbidity: a medical condition presented in patients with severe malaria, including chronic diseases, at least one of HIV, hypertension or diabetes mellitus,
- Mixed infection: infection of both *P*. *falciparum* and *P*. *vivax*
- Concomitant infection: includes community acquire pneumonia, aspiration pneumonia or meningitis
- Severe anemia: a hemoglobin level below <8 g/dl for males and <7 for females [21].
- Parasitaemia level: level of parasitaemia which reported semi-quantitatively (+1, +2, +3 or +4) [22].

### Sample size calculation and sampling technique

Sample size was determined using the single population proportion formula considering severe malaria mortality rate of 7.3% [23], and a 10% non-response rate (incomplete data).

n = $\frac{p(1-p){\left(z\alpha /2\right)}^{2}}{d^{2}}=\frac{0.073(1-0.073){\left(1.96\right)}^{2}}{0.05^{2}}$ = 103.98 + 10% non- response rate ≈115

Moreover, sample size determination was done using the possible predictors from the study reported 61.5% of severe malaria patients had neurologic manifestations in Northwest Ethiopia [23], giving the highest sample size.

n = $\frac{p(1-p){\left(z\alpha /2\right)}^{2}}{d^{2}}=\frac{0.615(1-0.615){\left(1.96\right)}^{2}}{0.05^{2}}$ = 363.83 + 10% non-response rate ≈ 400

Based on 2022 Health Management Information Systems registry of the hospital 745 severe malaria cases were attended the hospital during the study period, and computer-generated random patients were selected.

### Data collection procedure and tools

Data were collected using a structured questionnaire adapted from similar studies conducted elsewhere [24–26]. Socio-demographic characteristics, laboratory results, signs and symptoms, comorbidities, and management-related variables were incorporated into the data collection tool. All relevant data were collected from the medical records of severe malaria patients by referencing their Medical Record Number. The study’s outcomes were retrieved from the medical charts and/or death reports.

### Data processing and analysis

The data were assessed for completeness and recoded using Epidata version 3.1 and analyzed with SPSS version 20. Descriptive statistics were presented in frequency and percentage, and illustrated through tables. Mean and standard deviations were calculated to describe continuous variables. Bivariate and multivariate logistic regression analyses were conducted to determine associations between variables. Finally, statistical significance was declared at p < 0.05, with 95% confidence intervals.

### Ethical consideration

We obtained ethical clearance from the Institutional Review Board (IRB) of the University of Gondar (Reference number: 773/2015). A consent waiver was granted by the IRB since the study involved retrospective medical chart reviews. Code was used to hide the identities of study participants; therefore, the authors did not have access to their identity information.

## Results

### Socio demographic characteristic of the study participants

Of the total 400 study participants 17 (4.3%) of them were excluded from data analysis due to incomplete data. Three hundred eighty three (95.75%) of them were included including the non-response rate. The mean age of the study participants was 31.26 years ±12.46 SD. Almost half of the study participants were within 18–27 age groups. Majority of the study participants were males (56.66%), and rural residents (66.32%) (Table 1).

**Table 1:** Socio-demographic characteristics of the study participants at UoGCSH, Northwest Ethiopia (n = 383).

Socio-demographic characteristics		Frequency	Percentage (%)
Sex	Male	217	56.66
Age (in years)	18–27	187	48.8
	28–37	114	29.8
	38–47	25	6.5
	≥ 48	57	14.9
Residency	Urban	129	33.68
	Rural	254	66.32

### Clinical characteristics of the study participants during their admission

The commonly manifested and/or reported clinical features during the admission period were prostration, fever, impaired consciousness and convulsion in 96.87%, 63.71%, 25.85% and 16.97% of the study participants, respectively (Table 2).

**Table 2:** Clinical characteristics of the study participants at UoGCSH, Northwest Ethiopia (n = 383).

Clinical characteristics		Frequency	Percentage (%)
Fever	Yes	244	63.71
	No	139	36.29
Prostration	Yes	371	96.87
	No	12	3.13
Vomiting	Yes	208	54.31
	No	175	45.69
Impaired consciousness	Yes	99	25.85
	No	284	74.15
Convulsion	Yes	65	16.97
	No	318	83.03
Shock	Systolic BP*<80	10	2.5
	Systolic BP>80	390	97.5
	Diastolic BP>60	328	82
	Diastolic BP<60	72	18
Severe anemia	Yes	52	13
	No	348	87
Jaundice	Yes	52	13
	No	348	87
Creatinine level (mg/dl)	<3.0	319	87.2
	≥3.0	47	12.8
Spontaneous bleeding	Yes	22	5.7
	No	361	94.3
ARDS*	Yes	18	4.7
	No	365	95.3

* ARDS: Acute respiratory distress syndrome

*BP: Blood Pressure

### Risk factors related to severe malaria

Majority of the study participants 324 (84.60%) were referred from health facility. The mean time between the onset of the clinical manifestations and admission was 5.67 days with a standard deviation of 4.24. *Plasmodium falciparum* was the commonly identified parasite species. Three-fourth of the study participants had associated medical comorbidity (Table 3).

**Table 3:** Risk factors related to severe malaria among the study participants at UoGCSH, Northwest Ethiopia (n = 383).

Risk factors related to severe malaria		Frequency	Percentage (%)
Referral type	Self	59	15.40
	Health facility	324	84.60
Duration before admission (days)	1	66	17.2
	2–3	89	23.2
	>3	228	59.5
Species of malaria	*Plasmodium falciparum*	299	78.07
	*Plasmodium vivax*	52	13.58
	Mixed infection	32	8.36
Parasitemia level	≤2	311	81.20
	>2	72	18.80
Chronic disease	Yes	32	91.6
	No	351	8.4
Concomitant infections	Yes	118	30.8
	No	265	62.2
Pregnancy	Yes	49	12.8
	No	334	87.8

### Admission related factors

The mean hospital stay of the study participants was 7.79 days with standard deviation of 5.86. Most of the study participants 357 (93.21%) took intravenous Artesunate. More than half of the patients (51.44%) received antibiotics (Table 4).

**Table 4:** Admission related factors of severe malaria among the study participants at UoGCSH, Northwest Ethiopia (n = 383).

Admission related factors		Frequency	Percentage (%)
Hospital stays (days)	≤5	223	58.22
	6–14	116	30.29
	>14	44	11.49
Drugs taken	Artesunate	357	93.21
	Coartem	26	6.79
Blood transfusion	Yes	101	26.37
	No	282	73.63
Antibiotics	Yes	197	51.44
	No	186	48.56
Antipyretics	Yes	62	16.19
	No	321	83.81

### Treatment outcomes and associated factors

The magnitude of death among severe malaria patients was 42 (10.97%) (95% CI: 8.19–14.52), white the remaining 341 (89.03%) improved from an infection. In the bivariate logistic regression age, antibiotic usage before admission, parasitemia level, impaired consciousness, convulsion, jaundice, chronic disease, concomitant infections, and creatinine level were associated with severe malaria-related death at a p-value of ≤ 0.20. In the multivariate logistic regression model; there was a statistically significant association between age, impaired consciousness, convulsion, jaundice, parasitemia. The odds of death due to severe malaria was 7.04 folds among those above 48 years as compared with those of 18–27 years (AOR = 7.042, (95% CI; 2.2–21.8). Besides, the odds of severe malaria-related death were 4.2 folds among 28–37 years adults as compared with those of 18–27 years (AOR = 4.225 (95% CI; 1.5–11.6). The odds of death was 3.71 folds higher in patients with a parasitemia level of above 2 as compared with those with a parasitemia level of 2 and below (AOR = 3.717 (95% CI; 1.5–9.1). The odds of death among impaired consciousness patients were 3.4 times higher than those who had non-impaired consciousness (AOR = 3.401 (95% CI; 1.3–8.3). The odds of death among patients with jaundice was 3.2 folds higher than those who had no a clinical feature of jaundice (AOR = 3.282 (95% CI; 1.173–9.182). Moreover, the odd of death was 11.79 folds higher in patients who had a creatinine level of ≥ 3 as compared to those who had a creatinine level of < 3 (AOR = 11.793 (95% CI; 4.756–29.239) (Table 5).

**Table 5:** Bivariate and multivariate logistic regression analysis of factors associated with death among the study participants at UoGCSH, Northwest Ethiopia (n = 383).

Variables		Outcome		COR (95% CI)	*p*	AOR (95% CI)
		Improve	Death			
Age (years)	18–27	176	11	1		1
	28–37	97	17	2.804 (1.263,6.228)	0.005	4.225 (1.537–11.614
	38–47	21	4	3.048 (0.89–10.433)	0.062	4.018 (0.932–17.315)
	>48	46	11	3.826 (1.561–9.378)	0.001	7.042 (2.268–21.862)
Antibiotic usage admission	Yes	168	29	2.121 (1.083,4.154)	0.143	2.000 (0.791–5.057)
	No	173	13	1	1	1
Parasitemia level	≤2	283	28	1	1	1
	>2	57	15	2.660 (1.336–5.296)	0.004	3.717 (1.516–9.113)
Impaired consciousness	Yes	73	26	5.594 (2.880–10.865)	0.008	3.401 (1.383–8.366)
	No	267	17	1		1
Convulsion	Yes	49	16	3.519 (1.768–7.006)	0.049	2.742 (1.004–7.492)
	No	291	27	1		1
Jaundice	Yes	35	11	2.996 (1.388–6.464)	0.024	3.282 (1.173–9.182)
	No	305	32	1		1
Chronic disease	Yes	25	7	2.450 (0.990–6.064)	0.010	2.774 (0.818–9.410)
	No	315	36	1		1
Concomitant infections	Yes	93	25	3.689 (1.924–7.074)	0.1	2.2 (0.77–6.3)
	No	247	18	1		1
Creatinine level	< 3	298	21	1		1
	≥ 3	26	21	11.462 (5.548–23.679)	0.000	11.793 (4.756–29.239)

## Discussion

Malaria continues to be a significant cause of morbidity and mortality in tropical regions, including Ethiopia. In our study, the overall death among severe malaria patients was 10.97%; age, impaired consciousness, convulsion, parasitemia level of above two, elevated creatinine level and jaundice were the predictors for severe malaria related mortality.

In our study, the magnitude of death is consistent with the studies conducted in Mauritania (14.1%) [27] and Gambia (9.9%) [24]. However, the death rate in our study is higher than a study conducted at Arba-Minch Hospital in Ethiopia, where the death rate was 5.7% [28]. The difference might be due to the delayed presentation of our study participants to the health facility. The mean time between the onset of infection and admission was 5.67 days. This is relatively longer period of time when compared with Arba-Minch’s study which reported less than 5 days of presentation. The delayed treatment of severe malaria greatly heightens the risk of death, as the condition can swiftly progress to severe complications [29].

In contrast, our study death magnitude result is lower than the study conducted in the similar health facility (UoGCSH) about 18 years ago, 28.4% case fatality rate [23]. The variation could stem from differences in the study participants; the previous study focused on severe malaria patients meeting inclusion criteria for neurological manifestations. The 30% death prevalence among severe malaria patients in Sierra Leone is also higher than our study [30]. The difference might be attributed to differences in the age of study participants. The mean age in the Sierra Leone study is higher, at 58.5 years, compared to our study, which had a mean age of 31.26 years. Besides, our study elucidates the influence of age, revealing a statistically significant correlation with mortality. Specifically, individuals aged 48 years and above exhibited a 7.04 fold higher likelihood of death compared to those aged 18–27 years. This finding is in line with the studies conducted elsewhere [11, 31–34]. High burden in elder age group might be due to the increasing in comorbidities and decrease immunity in older age groups. (36). In addition, the rise in mortality with advancing age can be attributed to a higher prevalence of ARI among adults, which is associated with increased mortality. Acute tubular injury is implicated in the pathology of acute renal failure in severe malaria patients [35].

In addition, our study reported the elevated level of serum creatinine was significant risk factor severe malaria mortality. Previous studies revealed that AKI is one of the complications of severe malaria, accounting 40% of severe malaria patients, and 75% of severe malaria cases with AKI were died [36]. Similar result was reported in Brazil that non survivors of severe malaria were presented with the highest level of creatinine levels (39). Previous studies showed evidence of association between severe malaria, and kidney injury and some degree of liver abnormalities (40). These abnormalities often arise because patients with elevated creatinine levels are prone to developing complications associated with AKI, such as fluid overload, hyperkalemia, and acidosis. These complications, in turn, significantly elevate the risk of mortality among severe malaria patients.

The presence of jaundice, identified as a risk factor with a 3.3 fold increase in mortality, aligns with findings from studies in Mauritania [27] and Gambia [37], where jaundice was more prevalent among severe malaria patients. The association between jaundice and mortality in severe malaria may stem from its relation with severe liver dysfunction and subsequent hepatic failure. Elevated bilirubin levels, characteristic of jaundice, can accelerate systemic complications such as multi-organ dysfunction syndrome, exacerbating mortality risk. Jaundice often signifies advanced disease progression in severe malaria patients, reflecting the severity of *Plasmodium* infection and the subsequent systemic inflammatory response, ultimately leading to fatal outcomes.

Impaired consciousness as a death predictor in our study is consistent with a study conducted in Gambia [24, 33]. This is believed to occur due to the presence of parasitized red blood cells trapped in the small blood vessels of the brain, although some researchers suggest that impaired consciousness may also be influenced by metabolic factors and inflammatory mediators. The release of inflammatory mediators and metabolic disturbances can contribute to multi-organ dysfunction, ultimately leading to death in severe malaria cases. Besides, in our study patients presenting with convulsions had significantly higher odds of mortality compared to those without convulsions. Notably, there is limited literature examining convulsions as an independent variable. However, our findings align with previous study conducted in Gondar, Ethiopia, where approximately 59% of total deaths were attributed to cerebral malaria with associated convulsions [23].

The higher parasitaemia level in our study was also significantly associated with severe malaria related death. This finding is in agreement with study conducted in Asia [33]. Moreover, the level was parasitaemia was reported associated with acute kidney failure, which was discussed previously having a significant association [38]. The presence of many parasitized red blood cells which trap in the microvasculature could be result from cytoadherence. Higher parasite burdens indicate more severe infections, leading to widespread tissue damage and organ dysfunction. Micro-vascular sequestration worsens, impairing blood flow, hypoglycemia, acidosis and tissue oxygenation, contributing to complications like cerebral malaria and multi-organ failure. A robust immune response to higher parasitemia levels exacerbates systemic inflammation, further damaging tissues [39, 40].

## Limitations of the study

As the study is retrospective chart review, some variables are incomplete. Parasite (density) count not done as percentage which is the standard method; we used grading (semi-quantitative method). Variables such as body surface area and weight were not considered, even though they could influence the therapeutic effects of the treatment.

## Conclusion and recommendations

Our study revealed a significant number of deaths related to severe malaria, which is concerning for stakeholders as the country strives towards malaria elimination. Achieving a malaria-free Ethiopia necessitates comprehensive interventions aimed at reducing the severity of malaria and associated mortality rates. Early identification and intensive care provision for severe malaria patients exhibiting older age, impaired consciousness, convulsions, parasitemia level greater than 2+, creatinine level ≥3mg/dl, and jaundice are crucial. Ensuring the availability of malaria diagnostic and therapeutic agents at all times at all facilitates prompt recognition of important predictors, aiding in the prevention of severe malaria-related mortality. The previously mentioned predictors of severe malaria death are interrelated factors that contribute to the escalation of illness severity and ultimately lead to fatal outcomes. These variables often coexist in patients, exacerbating the pathological progressions underlying severe malaria. Understanding the complex interplay between these predictors is crucial for improving risk stratification and developing targeted interventions to mitigate the progression of severe malaria and reduce mortality rates.
